# A bibliometric analysis of the published studies in ciprofol research

**DOI:** 10.1097/MD.0000000000043567

**Published:** 2025-07-25

**Authors:** Wei Wu, Yanting Sun, Zhaoting Li, Yuntai Yao

**Affiliations:** a Department of Anesthesiology, Baoji High-Tech Hospital, Shaanxi, China; b Department of Anesthesiology, Fuwai Hospital, National Center for Cardiovascular Diseases, Peking Union Medical College and Chinese Academy of Medical Sciences, Beijing, China; c Center of Outcomes Research, Department of Anesthesiology, Critical Care and Pain Medicine, University of Texas, Houston, TX; d Outcomes Research Consortium, Houston, TX.

**Keywords:** bibliometric analysis, ciprofol, research trends

## Abstract

**Objective:**

Ciprofol (HSK-3486), a novel intravenous anesthetic with structural similarities to propofol, has shown promising pharmacokinetic (PK) and pharmacodynamic (PD) profiles; however, clinical data regarding its efficacy and safety remain limited. Because research on ciprofol is still in its infancy, this study uses bibliometric methods to examine the published literature and to highlight research trends, key topics, and future directions for its clinical applications.

**Methods:**

We searched the Web of Science Core Collection (WoSCC) for English-language articles and reviews on ciprofol published from 2017 – the year it was first reported – through August 28, 2024. Two authors independently screened titles and abstracts for eligibility, discrepancies were resolved in consultation with a senior reviewer. The data, including publication details and citation metrics, were extracted and organized for analysis. We visualized the results with CiteSpace, GraphPad Prism, and Microsoft Excel.

**Results:**

A total of 62 studies were included. Annual publications rose by 50% from 2021 to 2022 and by 83% from 2022 to 2023. China dominated the field, contributing 59 articles (95.16% of the total). Sichuan University and Liu X. were the most prolific institution and author, respectively. *BMC Anesthesiology*, *Drug Design, Development and Therapy*, *Frontiers in Pharmacology*, and *Journal of Clinical Anesthesia* were the 4 leading journals publishing ciprofol research. *Frontiers in Pharmacology* received the most citations (n = 71) and achieved an H-index of 4. *Anesthesiology* carried the highest 2022 impact factor (9.1), whereas *Anesthesia and Analgesia* accrued 48 citations. Safety, pharmacokinetics, and anesthesia were the most frequently studied aspects, whereas “critically ill patients,” “injection,” and “pain” emerged as recent research hotspots. Temporal analysis showed that key terms shifted from “pharmacokinetics” in 2021 to “general anesthesia” by 2022, while themes such as “critically ill patients” remained consistently relevant.

**Conclusion:**

This bibliometric analysis provides a comprehensive overview of ciprofol research, identifies key contributors, and reveals critical insights and gaps in the literature. Our findings underscore ciprofol’s safety, efficacy, and optimal clinical use while highlighting current hotspots and challenges that should guide future research.

## 1. Introduction

Ciprofol (HSK-3486), developed by Haisco Pharmaceutical Group Co., Ltd. (Chengdu, China) and first reported in 2017,^[1]^ is a novel 2,6-disubstituted phenol derivative that acts as a structural analogue of propofol and a potentiator of the γ-aminobutyric acid type A (GABAA) receptor. The addition of an R-chiral center and a cyclopropyl group improves its PK characteristics and PD responses,^[2,3]^ resulting in well-defined absorption, distribution, metabolism, and excretion (ADME) profiles.^[4]^ On December 15, 2020, the China National Medical Products Administration (NMPA) approved ciprofol for sedation during gastrointestinal endoscopy. Subsequently, it was authorized for bronchoscopy-related sedation,^[5]^ and for the induction and maintenance of general anesthesia.^[6]^ Most recently, the NMPA approved ciprofol for sedation in intensive-care settings.^[7]^ According to ClinicalTrials.gov, a phase III trial evaluating ciprofol for sedation and anesthesia in outpatient gynecologic surgery has been completed in China, and the indication is under regulatory review.^[8]^ Ciprofol is therefore expected to broaden its clinical applications further.

As a novel intravenous anesthetic structurally analogous to propofol, ciprofol exhibits favorable PD properties, including rapid onset and offset during induction of general anesthesia. Phase 1a and 1b trials in Australia^[9]^ and China^[10]^ showed that single doses of 0.1 to 0.9 mg kg^−1^ were well tolerated and safe. Subsequent multi-center phase 2 and 3 studies compared ciprofol with propofol for induction and maintenance of general anesthesia in elective surgery^[6,11–14]^ and for sedation during mechanical ventilation in the ICU,^[7]^ gastroscopy,^[2]^ colonoscopy,^[15]^ fiber-optic bronchoscopy,^[5]^ endoscopic retrograde cholangiopancreatography (ERCP),^[16]^ and hysteroscopy.^[17]^ In a multi-center phase 2 trial, Teng et al^[15]^ showed that ciprofol 0.4 to 0.5 mg kg^−1^ provided sedation equivalent to propofol 2.0 mg kg^−1^ during colonoscopy, with comparable safety and markedly less injection pain, likely owing to its lower free-drug concentration. Furthermore, ciprofol appears to confer more stable hemodynamics and fewer adverse events than propofol.^[4]^ Yang et al^[18]^ reported that ciprofol protects against isoproterenol-induced myocardial infarction by reducing cardiac oxidative stress, inflammation, and cardiomyocyte apoptosis. A phase 3 study^[19]^ further showed that ciprofol 0.2 to 0.4 mg kg^−1^ reliably induced general anesthesia in older patients undergoing major noncardiac surgery, with a lower incidence of hypotension, rare injection pain, and no serious adverse events.

Despite encouraging preclinical and clinical data,^[20,21]^ clinical trials of ciprofol – China’s first Class 1 innovative intravenous anesthetic – remain scarce. Its efficacy and safety across diverse surgical procedures and patient populations therefore require further elucidation. To our knowledge, this is the first bibliometric study of ciprofol, aimed at mapping research trends, hot topics, and future directions. Specifically, we examined annual publication trends, the most productive institutions, the evolution of high-frequency keywords and hotspots, and patterns of international collaboration and citation impact. Our findings are intended to inform future ciprofol research and guide its clinical development.

## 2. Methods

### 2.1. Sources of data and search strategy

**Figure 1.**
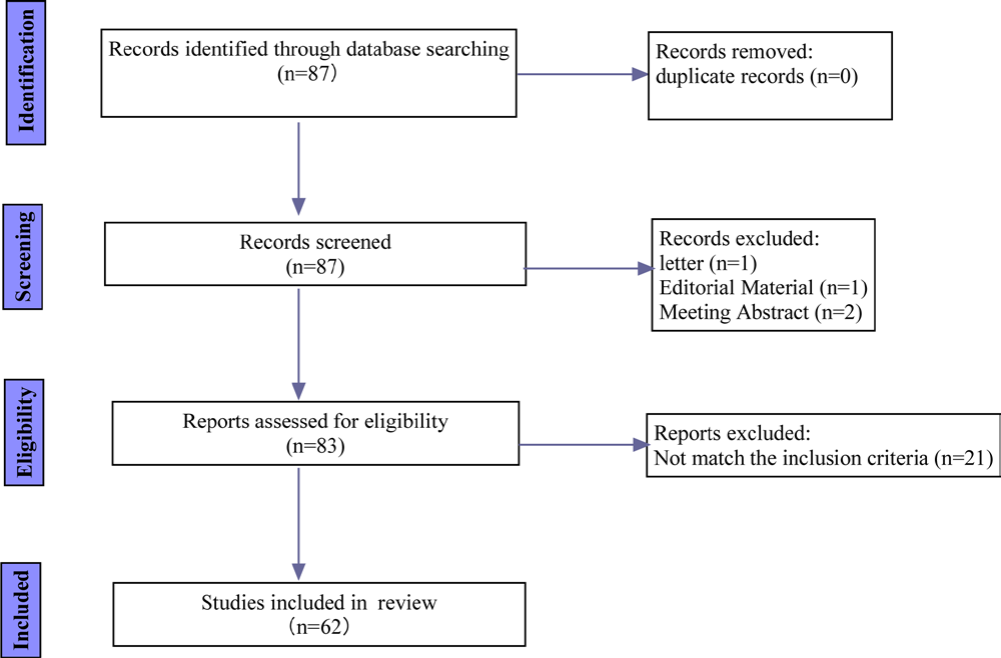
BIBLO flow diagram of study selection. BIBLO = bibliometric.

The Web of Science (WoS), known for its broad disciplinary coverage, comprehensive citation indexing, and rich analytical indicators, serves as a primary database for bibliometric analysis. We extracted publication data from the Web of Science Core Collection (WoSCC), which offers extensive coverage and detailed statistics for bibliometric research.^[22]^ To minimize bias, we performed all searches, data extractions, and downloads on the same day. We focused on articles and reviews published from 2017 – when “ciprofol” was first reported – through August 28, 2024, using the search strategy: “TS = (ciprofol or HSK3486 or novel 2,6-disubstituted phenol derivatives).” Only English-language human studies were included; the selection process is depicted in Figure 1.

### 2.2. Study selection and data extraction

Two authors (WW and YTS) independently screened titles and abstracts, then retrieved full texts from WoSCC for eligibility; disagreements were resolved in consultation with a senior reviewer (YTY). Specifically, we calculated the Cohen kappa value, which quantifies the agreement between the reviewers. The resulting kappa value was 0.94, indicating very high consistency between the reviewers. Data collection followed these criteria: database, Web of Science Core Collection; citation indexes, science citation index expanded (SCI-E) and social science citation index; search period, January 1, 2017 to August 28, 2024; language, English; keywords, “ciprofol,” “HSK3486,” or “Novel 2,6-Disubstituted Phenol Derivatives”; document types, articles and reviews; export format, plain text; and species, human studies only. Extracted variables included title, authors, institution, country, journal (with 2023 impact factor [IF]), publication year, citation count, and H-index.

### 2.3. Strategy for data synthesis

Publications and their cited references were exported as plain-text files for bibliometric analysis and visualization. We used CiteSpace (version 6.3 R1), GraphPad Prism (version 10.0), and Microsoft Excel 2007 for analysis. Excel handled data organization, whereas GraphPad Prism generated line graphs of annual publications, citations, and H-index values. CiteSpace, a Java-based program developed by Chen (2004),^[23]^ generated timeline maps and detected keyword bursts. In the visualizations, nodes represent countries, institutions, authors, or journals, clustered according to collaboration, with node size indicating the number of publications. The thickness of the lines connecting nodes reflects link strength (LS), representing the strength of cooperation, while total LS (TLS) indicates overall collaboration levels.^[22]^ For the keyword analysis, irrelevant terms were removed and synonyms merged to improve clarity. Mean silhouette values (S > 0.7) and modularity (Q > 0.3) confirmed that the CiteSpace clusters were well defined.^[24]^

## 3. Results

### 3.1. Study selection and characteristics

**Figure 2.**
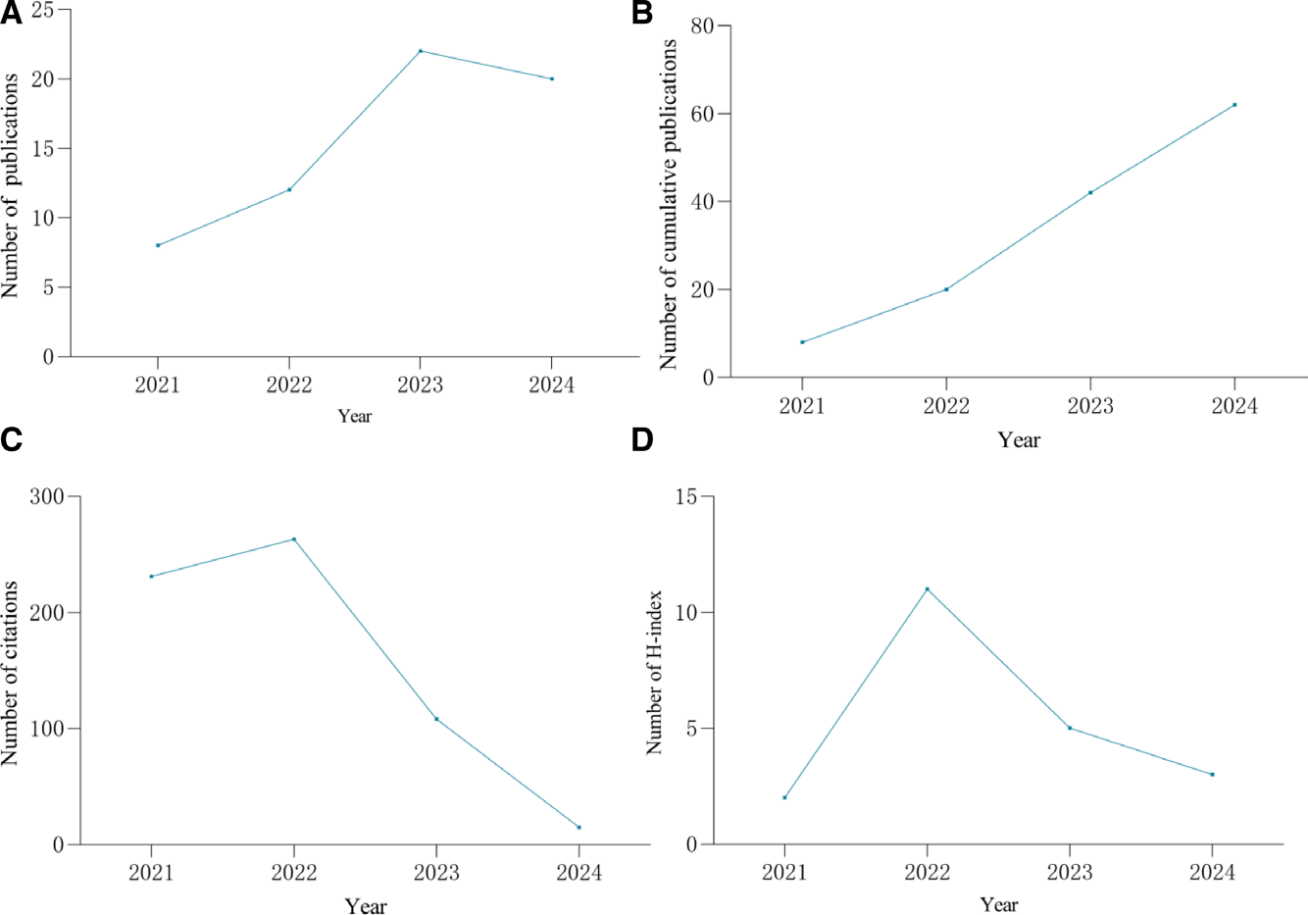
(A) The annual number of publications; (B) the annual number of cumulative publications; (C) the annual number of citations of the publications; (D) the annual H-index values of the publication.

The literature search and selection process are shown in Figure 1. Initially, 87 records were identified, and 62 met the inclusion criteria. Annual publication counts for 2017 to 2024 are displayed in Figure 2A. Annual output increased overall, with a marked acceleration between 2021 and 2023. Specifically, publication numbers rose by 50 % between 2021 and 2022 and by a further 83.33 % between 2022 and 2023. Although 2024 showed a slight decline, the overall upward trend persisted up to the search cutoff. Cumulative publications rose steadily from 2017 to 2024 (Fig. 2B), indicating growing interest in ciprofol. Citations peaked in 2022 (Fig. 2C), averaging 155 that year, whereas the annual H-index climbed from 2 in 2021 to 11 in 2022 (Fig. 2D).

### 3.2. Distribution of publication by country and institution

**Figure 3.**
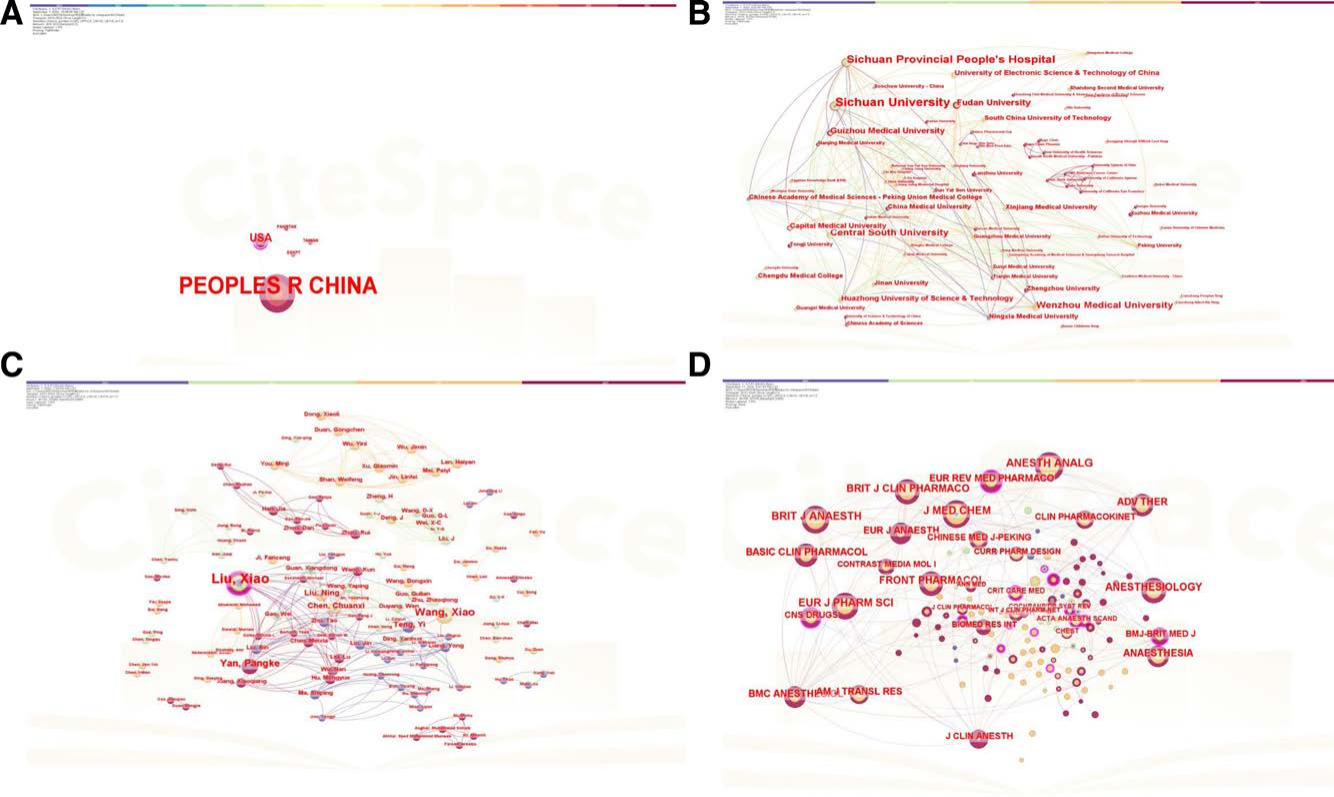
The co-authorship network map of countries (A) institutions (B) authors (C and D) The co-citation network map of journal.

Figure 3A presents the country-level co-authorship network. Overall, 5 country and 3 cooperation instances were presented. China led with 59 articles, representing 95.16 % of all publications. The United States followed with 6 articles (8.57 %), whereas Egypt and Pakistan contributed one each (1.43 %). Three discrete international collaboration links were also identified. Among them, the United States showed the strongest collaboration (LS = 3). China also accrued the most citations (n = 608) and the highest H-index (17). Figure 3B depicts an institutional co-authorship network of 74 institutions with 204 collaboration links. Sichuan University formed the densest network, with a TLS of 21. The top 30 most productive institutions are listed in Table 1. Sichuan University produced 13 papers (20.97 %), followed by Sichuan Provincial People’s Hospital with 11 (17.74 %) and Wenzhou Medical University with 7 (11.29 %). Additionally, Sichuan University accrued the highest citation count (n = 285) and the greatest H-index (5).

**Table 1:** The top 30 productive institutions regarding ciprofol in clinical setting from 2021 to 2024.

Rank	Institution	Count	Percentage	Country	Year	Total citations	H-Index
1	Sichuan University	13	20.97	China	2021	285	5
2	Sichuan Provincial People’s Hospital	11	17.74	China	2021	261	5
3	Wenzhou Medical University	7	11.29	China	2021	173	5
4	Central South University	7	11.29	China	2021	195	5
5	Fudan University	6	9.68	China	2022	63	3
6	Guizhou Medical University	5	8.07	China	2022	82	0
7	University of Electronic Science and Technology of China	4	6.45	China	2022	55	3
8	Capital Medical University	4	6.45	China	2022	36	2
9	Huazhong University of Science and Technology	4	6.45	China	2021	141	4
10	Chengdu Medical College	3	4.84	China	2022	47	1
11	Chinese Academy of Medical Sciences – Peking Union Medical College	3	4.84	China	2021	123	3
12	South China University of Technology	3	4.84	China	2022	61	2
13	Ningxia Medical University	3	4.84	China	2021	103	1
14	Xinjiang Medical University	3	4.84	China	2022	71	3
15	Jinan University	3	4.84	China	2022	82	3
16	Zhengzhou University	3	4.84	China	2022	30	0
17	China Medical University	3	4.84	China	2022	28	0
18	Guangzhou Medical University	2	3.23	China	2022	32	2
19	Shandong Second Medical University	2	3.23	China	2023	8	1
29	Sun Yat-Sen University	2	3.23	China	2021	44	2
21	Tianjin Medical University	2	3.23	China	2022	40	0
22	Lanzhou University	2	3.23	China	2024	1	0
23	Guangxi Medical University	2	3.23	China	2022	17	2
24	Zunyi Medical University	2	3.23	China	2022	74	2
25	Soochow University	2	3.23	China	2021	67	2
26	Xuzhou Medical University	2	3.23	China	2024	1	0
27	Tongji University	2	3.23	China	2024	1	0
28	Chinese Academy of Sciences	2	3.23	China	2023	0	0
29	Peking University	2	3.23	China	2022	34	2
30	Nanjing Medical University	2	3.23	China	2022	29	0

Counts refers to the number of published papers. Percentage refers to the proportion of the number of published papers. Total Citations refers to the overall number of citations received by all publications. H-index measures the productivity and citation impact of an author, with an H-index of h indicating h papers with at least h citations each.

### 3.3. Analysis of authors publications

In total, 122 authors contributed to the dataset, generating 285 collaboration links. The 21 most prolific authors are listed in Table 2. Liu X. (Haisco Pharmaceutical Group) led with 11 papers, followed by Wang X. (Sichuan University, n = 6) and Yan P.K. (Haisco Pharmaceutical Group, n = 5); all 3 authors are based in China. Liu X. also garnered the most citations, whereas Wang X. achieved the highest H-index. The author-level collaboration network is visualized in Figure 3C. Liu X. maintained the most collaboration links (TLS = 26).

**Table 2:** The top 21 most productive authors regarding ciprofol in clinical setting from 2021 to 2024.

Rank	Authors	Counts	Year	Percentage	Total citations	H-index	Institutions
1	Liu, Xiao	11	2021	17.74	214	5	Haisco Pharmaceut Grp
2	Wang, Xiao	6	2021	9.68	197	6	Sichuan University
3	Yan, Pangke	5	2021	8.06	90	3	Haisco Pharmaceut Grp
4	Teng, Yi	3	2021	4.84	129	3	Sichuan University
5	Chen, Chuanxi	3	2021	4.84	49	3	Sun Yat-Sen University
6	Liu, Ning	3	2021	4.84	23	1	Sun Yat-Sen University
7	Liang, Yong	2	2021	3.23	94	2	Haisco Pharmaceut Grp
8	Liu, Bin	2	2021	3.23	94	2	Sichuan University
9	Liu, Jin	2	2021	3.23	94	2	Sichuan University
10	Ma, Shiping	2	2021	3.23	84	2	Haisco Pharmaceut Grp
11	Guo, Qulian	2	2022	3.23	74	2	Xiangya Hospital
12	Wang, Yaping	2	2022	3.23	74	2	Xiangya Hospital
13	Ouyang, Wen	2	2022	3.23	74	2	Xiangya Hospital
14	Zhu, Zhaoqiong	2	2022	3.23	74	2	Zunyi Medical University
15	Gao, Wei	2	2021	3.23	44	2	Haisco Pharmaceut Grp
16	Liu, J	2	2022	3.23	44	2	Sichuan University
17	Guan, Xiangdong	2	2021	3.23	44	2	Sun Yat-Sen University
18	Guo, Q-L	2	2022	3.23	44	2	Xiangya Hospital
19	Wei, X-C	2	2022	3.23	44	2	Sichuan Provincial People’s Hospital
20	Zheng, H	2	2022	3.23	44	2	Xinjiang Medical University
21	Wang, D-X	2	2022	3.23	44	2	Peking University

Counts refers to the number of published papers. Percentage refers to the proportion of the number of published papers. Total citations refers to the overall number of citations received by all publications. H-index measures the productivity and citation impact of an author, with an H-index of h indicating h papers with at least h citations each.

### 3.4. Analysis of journal distribution

Thirty-six journals published ciprofol studies, and the 10 most productive accounted for 58.06 % of all articles (Table 3). Notably, *BMC Anesthesiology, Drug Design, Development and Therapy, Frontiers in Pharmacology*, and *the Journal of Clinical Anesthesia* were the 4 leading outlets. *Frontiers in Pharmacology* received the most citations (n = 71) and recorded an H-index of 4, whereas *Advances in Therapy* showed the highest mean citations per article. IF remains a key indicator of journal influence.^[24]^ Some high-impact journals with an IF of 7 or higher published literature in this field, such as *Anesthesiology* (9.1), *American Journal of Gastroenterology* (8), *Critical-Care Medicine* (7.7), *Chinese Medical Journal* (7.5), and *CNS Drugs* (7.4). The journal co-citation network is illustrated in Figure 3D. The 4 most co-cited journals were *Anesthesia and Analgesia* (48), the *British Journal of Anesthesia* (47), the *Journal of Medicinal Chemistry* (44), and the *European Journal of Pharmaceutical Sciences* (42).

**Table 3:** The top 15 most productive journals regarding ciprofol from 2017 to 2024.

Rank	Journal title	Records	IF (2023)	Total citations	Average citation	H-index
1	BMC Anesthesiol	7	2.3	34	4.86	1
2	Drug Des Devel Ther	6	4.7	10	1.76	1
3	Front Pharmacol	6	4.4	71	11.83	4
4	J Clin Anesth	4	5	24	6.00	3
5	Eur Rev Med Pharmacol Sci	3	/	44	14.67	0
6	Adv Ther	2	3.4	52	26.00	2
7	Anesth Analg	2	4.6	2	1.00	0
8	Eur J Anesthesiol	2	4.2	31	15.50	2
9	Fronti Med	2	3.1	2	1.00	0
10	Heliyon	2	3.4	1	0.50	0
11	Anesthesiology	1	9.1	4	4.00	1
12	Am J Gastroenterol	1	8	0	0.00	0
13	Crit Care Med	1	7.7	5	5.00	1
14	Chin Med J	1	7.5	27	27.00	1
15	CNS Drugs	1	7.4	34	34.00	1

Records refers to the number of published papers. IF (2023) refers to the impact factor of the journal in the year 2023. Total citations refers to the overall number of citations received by all publications. H-index measures the productivity and citation impact of an author, with an H-index of h indicating h papers with at least h citations each.

IF = impact factor.

### 3.5. Analysis of highly cited publications

Table 4 lists the 10 most-cited studies, 6 of which originated from West China Hospital, Sichuan University. The article “Efficacy and Safety of Ciprofol for Sedation/Anesthesia in Patients Undergoing Colonoscopy: Phase IIa and IIb Multicenter Clinical Trials,” published in the *European Journal of Pharmaceutical Sciences* in 2021, received the most citations (n = 82, WoS) and therefore ranks first. Among the top-cited papers, five explored ciprofol PK/PD, 2 assessed its efficacy and safety for colonoscopy sedation, and the remainder investigated use during mechanical ventilation, bronchoscopy, or gynecologic surgery.

**Table 4:** The top 10 most-cited references regarding ciprofol in clinical setting from 2021 to 2024.

Rank	Title	Authors	Journal	Institution	Citations
1	Efficacy and safety of ciprofol for the sedation/anesthesia in patients undergoing colonoscopy: Phase IIa and IIb multi-center clinical trials	Yi Teng, Jin Liu, et al	Eur J Pharm Sci	Sichuan University, China	289
2	Mass balance, pharmacokinetics and pharmacodynamics of intravenous HSK-3486, a novel anesthetic, administered to healthy subjects	Yicong Bian, Liyan Miao, et al	Br J Clin Pharmacol	the First Affiliated Hospital of Soochow University, Suzhou, China	277
3	Comparison of ciprofol (HSK-3486) versus propofol for the induction of deep sedation during gastroscopy and colonoscopy procedures: A multi-center, non-inferiority, randomized, controlled phase 3 clinical trial	Junxiang Li, Yunxia Zuo, et al	Basic Clin Pharmacol Toxicol	Sichuan University, China	73
4	Sedation Effects Produced by a Ciprofol Initial Infusion or Bolus Dose Followed by Continuous Maintenance Infusion in Healthy Subjects: A Phase 1 Trial	Chao Hu, Jia Miao, et al.	Adv Ther	Sichuan University, China	218
5	Pharmacokinetic and pharmacodynamic properties of ciprofol emulsion in Chinese subjects: a single center, open-label, single-arm dose-escalation phase1 study	Yi Teng, Meng-Chan Ou, et al	Am J Transl Res	Sichuan University, China	227
6	Safety, Pharmacokinetics, and Pharmacodynamics of a Single Bolus of the γ-aminobutyric Acid (GABA) Receptor Potentiator HSK-3486 in Healthy Chinese Elderly and Non-elderly	Xiaojiao Li, Hushan Wang, et al	Front Pharmacol	Jilin University, Jilin, China	112
7	Effects of ciprofol for the induction of general anesthesia in patients scheduled for elective surgery compared to propofol: a phase 3, multi-center, randomized, double-blind, comparative study	X. Wang, Y-X Zuo, et al	Eur Rev Med Pharmacol Sci	Sichuan University, China	97
8	Efficacy and safety of HSK-3486 for anesthesia/sedation in patients undergoing fiberoptic bronchoscopy: a multi-center, double-blind, propofol-controlled, randomized, phase 3 study	Zhen Luo, Hong Tu, et al	CNS Drugs	Sichuan University, China	105
9	Safety and efficacy of ciprofol versus propofol for sedation in intensive-care unit patients with mechanical ventilation: a multi-center, open-label, randomized, phase 2 trial	Yongjun Liu, Xiangdong Guan, et al	Chin Med J	The First Affiliated Hospital, Sun Yat-sen University, Guangzhou	86
10	The eﬃcacy and safety of ciprofol use for the induction of general anesthesia in patients undergoing gynecological surgery: a prospective randomized control	Ben‑zhen Chen, Bi‑ying Yuan, et al	BMC Anesthesiol	Sichuan Provincial Women’s and Children’s Hospital	87

### 3.6. Keyword analysis of research hotspots

**Figure 4.**
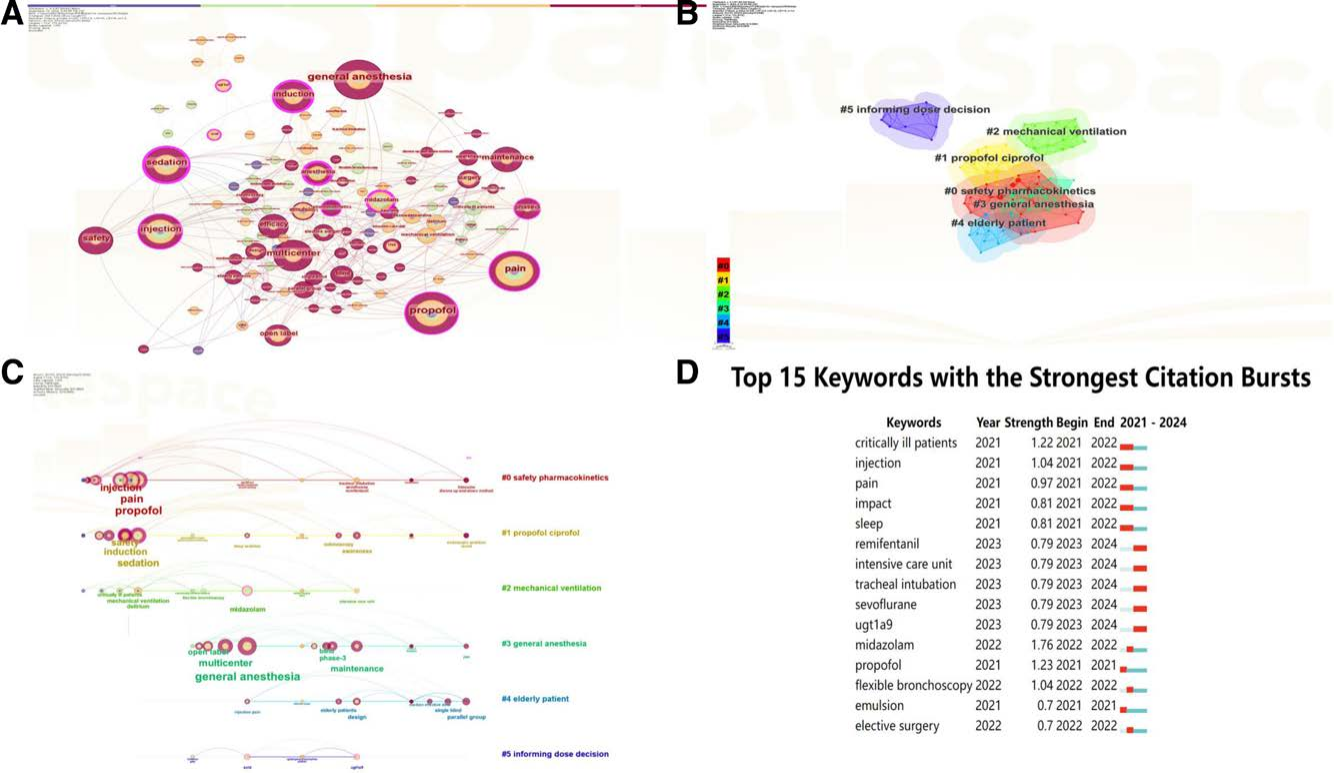
The network map (A) cluster (B) and timeline (C) map of keyword co-occurrence. (D) The top 15 keywords with the strongest citation bursts.

Keyword co-occurrence analysis helps identify prevalent research topics. The co-occurrence keyword network is displayed in Figure 4A. The 10 most frequent keywords were “general anesthesia,”“propofol,” “pain,” “injection,” “sedation,” “multicenter,” “safety,” “induction,” “maintenance,” and “efficacy.” Figure 4B groups all keywords into 6 clusters. The largest cluster (red) centers on safety-related pharmacokinetics and includes “injection,” “pain,” and “anesthesia.” The yellow cluster links propofol and ciprofol, encompassing “sedation,” “induction,” “safety,” “efficacy,” and “awareness.” The green cluster relates to mechanical ventilation and includes “delirium,” “midazolam,” and “mechanical ventilation.” The blue cluster represents general anesthesia, containing “general anesthesia,” “multicenter,” “maintenance,” “open label,” and “elective surgery.” The gray cluster concerns elderly patients and features “design,” “parallel group,” “single blind,” and “injection pain.” The purple cluster addresses dose-decision support and includes “common polymorphism,” “propofol injection,” and “inhibition.” Figure 4C depicts keyword evolution over time (CiteSpace: *Q* = 0.5602; *S* = 0.8843). In 2021, prominent terms were “propofol,” “ciprofol,” “safety-related PKs,” and “mechanical ventilation,” whereas “general anesthesia” emerged as a major focus by 2024. By 2024, key topics encompassed elderly patients, safety PKs, propofol, ciprofol, and general anesthesia. Figure 4D shows keyword burst strengths: “critically ill patients” displayed the strongest burst (1.22), followed by “injection” (1.04) and “pain” (0.97). Notably, bursts for “remifentanil,” “intensive care unit,” “tracheal intubation,” “sevoflurane,” and “UGT1A9” persisted into 2024, indicating that these remain active hotspots.

## 4. Discussion

Our data show a marked rise in annual publications from 2021 onward, coinciding with the China NMPA’s approval of ciprofol for gastrointestinal endoscopy sedation on December 15,2020. Notably, both citations and H-index peaked in 2022, reflecting heightened academic interest and impact. This surge likely reflects intensified research activity and greater recognition of ciprofol’s clinical relevance. The modest decline in citations and H-index for 2024 probably relates to the proximity of the data-collection cutoff. These findings underscore an evolving research landscape and the need for continued studies to clarify ciprofol’s applications and benefits.

The distribution of publications underscores China’s preeminence, evidenced by its leading collaboration links, article count, citations, and the highest H-index. This dominance implies that China is not only producing most of the research but also driving development and commercialization, likely because the drug is manufactured domestically. The 3 detected international links – most prominently with the United States – indicate some cross-border engagement. Nevertheless, the overall paucity of substantial international collaboration remains concerning. Such limited interplay may impede the diversification of perspectives and ultimately constrain innovation. Strengthening international partnerships and encouraging input from under-represented countries will be vital for a balanced research ecosystem.

Sichuan University and Liu X. emerged as the most influential institution and author, respectively, reflecting their productivity and impact. The participation of several authors from Sichuan University and Haisco Pharmaceutical Group points to a collaborative environment conducive to sharing resources and expertise. Sichuan University’s TLS of 21 denotes a robust network, critical for tackling complex research questions. Contributions from Sichuan Provincial People’s Hospital and Wenzhou Medical University further underscore strong regional collaboration and a concentrated domestic research environment. Broadening this network internationally could enhance the potential for breakthrough discoveries.

The most impactful journals were *BMC Anesthesiology*; *Drug Design, Development and Therapy*; *Frontiers in Pharmacology*; and t*he Journal of Clinical Anesthesia*. *BMC Anesthesiology* led in volume and H-index, Frontiers in Pharmacology accrued the most citations, *Advances in Therapy* showed the highest mean citations per paper, and Anesthesiology carried the greatest IF. The co-citation network underscores this interconnectivity, positioning *Anesthesia and Analgesia*, the *British Journal of Anesthesia*, the *Journal of Medicinal Chemistry*, and the *European Journal of Pharmaceutical Sciences* as key nodes. These insights help delineate the evolving trajectory of ciprofol scholarship.

Analysis of the 10 most-cited papers underscores their substantial influence on anesthetic practice. The top-cited article evaluated ciprofol for colonoscopy sedation and showed that 0.4 to 0.5 mg kg^−^¹ matched the success and safety of propofol 2.0 mg kg^−^¹ without significant adverse events. Teng et al^[15]^ confirmed this range in phase IIa/IIb trials, reporting 100 % procedural success. Bian et al^[4]^ investigated PK/PD in 6 healthy volunteers given 0.4 mg kg^−^¹, demonstrating rapid onset and good tolerability; the glucuronide metabolite M4 accounted for 79.3% of plasma exposure, and only mild hypotension occurred. Subsequent studies by Teng et al^[10]^ and Wang et al^[6]^ confirmed that ciprofol emulsion induces short-term anesthesia with less injection pain and respiratory depression and with more stable hemodynamics than propofol. Hu et al^[25]^ showed that continuous infusion provides effective sedation for up to 12h with safety comparable to propofol. Li et al^[26]^ demonstrated effective dosing in elderly patients with outcomes comparable to propofol, reinforcing ciprofol’s potential in this population. Li et al^[2]^ and Liao et al^[27]^ further showed fewer adverse reactions during gastroscopy, colonoscopy, and gynecologic procedures, supporting ciprofol as an effective propofol alternative. Luo et al^[5]^ reported 100 % procedural success and fewer adverse events during bronchoscopy. In mechanically ventilated patients, Liu et al^[7]^ found comparable sedation quality and tolerability to propofol, with similar rates of treatment-emergent events. Taken together, current evidence suggests that ciprofol matches propofol’s efficacy while offering a lower adverse-event burden, warranting further clinical adoption and investigation.

Keyword co-occurrence analysis offers additional insight into the ciprofol research landscape. Prominent terms – “general anesthesia,” “propofol,” and “safety” – highlight the field’s emphasis on anesthetic practice and patient safety. The largest cluster, focused on safety-related pharmacokinetics, signals growing interest in ciprofol metabolism and its clinical impact. Keywords such as “injection” and “pain” suggest efforts to optimize administration routes and improve patient comfort. The yellow cluster, linking propofol and ciprofol, reflects ongoing comparative studies that align with personalized-medicine goals. The green cluster associates mechanical ventilation with “delirium,” underscoring the importance of anesthetic management in critically ill patients. The blue cluster, centered on general anesthesia in multi-center elective surgery, points to the need for standardized protocols. The gray cluster highlights elderly patients, reflecting demographic shifts and the necessity to tailor anesthetic practice to their distinct profiles. Temporal analysis shows “general anesthesia” emerging as a key term in 2022, signaling a shift toward refined protocols and improved surgical outcomes. The sustained prominence of terms relating to elderly patients and safety-PK in 2024 indicates that these themes will remain central. Burst analysis highlights “critically ill patients” as a growing focal area and persistent bursts for “remifentanil” and “sevoflurane,” illustrating the dynamic evolution of anesthetic practice. Overall, continued exploration of safety, pharmacokinetics, and patient-centered care will be essential to advance anesthetic practice and improve outcomes across surgical and critical-care settings.

This study has several limitations. First, the search was restricted to WoSCC, which may have yielded an incomplete overview of the literature. Nonetheless, WoSCC is one of the largest and most comprehensive sources for bibliometrics and likely provides an adequate picture of the field. Second, inclusion was limited to English-language studies, potentially excluding relevant work in other languages. Because ciprofol is novel, some relevant studies may be registered but not yet published. A major limitation is that the analysis mainly includes studies from China, where ciprofol was developed and first approved. This concentration of research may limit the generalizability of the findings. Although some international collaborations were identified, the focus on Chinese studies reflects ciprofol’s early-stage development. Future research should include a broader global perspective as its clinical adoption grows. Finally, publication bias and reliance on published data may have obscured ongoing or unpublished research.

In summary, this bibliometric and visual analysis provides a comprehensive overview of ciprofol’s clinical research landscape. We identified key contributors – countries, institutions, authors, and journals – thereby revealing critical insights and gaps in the literature. Our findings emphasize ciprofol’s safety, efficacy, and optimal use as a sedative-anesthetic, while delineating its advantages and remaining challenges. The work also maps current hotspots and challenges, offering a valuable reference for future investigations.

## Acknowledgments

All authors are very grateful to reviewers and editors for their help and suggestions. They also like to thank the members of the Evidence in Cardiovascular Anesthesia (EICA) Group for their invaluable contributions and support. The members of the EICA Group are: Wei Wu, Yanting Sun, Zhaoting Li, and Yuntai Yao.

## Author contributions

**Conceptualization:** Yanting Sun, Wei Wu, Zhaoting Li, Yuntai Yao.

**Methodology:** Wei Wu, Zhaoting Li.

**Software:** Wei Wu.

**Supervision:** Yuntai Yao.

**Visualization:** Yanting Sun.

**Writing – original draft:** Yanting Sun, Wei Wu, Zhaoting Li.

**Writing – review & editing:** Yuntai Yao.
